# Distinctive Temporal Profiles of Interferon-Stimulated Genes in Natural Infection, Viral Challenge, and Vaccination

**DOI:** 10.3390/v17081060

**Published:** 2025-07-29

**Authors:** Hongxing Lei

**Affiliations:** 1 China National Center for Bioinformation, Beijing 100101, China; leihx@big.ac.cn; Tel.: +86-010-84097276; 2 Beijing Institute of Genomics, Chinese Academy of Sciences, Beijing 100101, China; 3 Medical School, University of Chinese Academy of Sciences, Beijing 101408, China

**Keywords:** COVID-19, vaccination, interferon signaling, GBP gene cluster, HLA-D gene cluster

## Abstract

Interferon (IFN) signaling plays vital roles in host defense against viral infection. However, a variety of observations have been reported in the literature regarding the roles of IFN signaling in COVID-19. Thus, it would be important to reach a clearer picture regarding the activation or suppression of IFN signaling in COVID-19. In this work, regulation of marker genes for IFN signaling was examined in natural infection, viral challenge, and vaccination based on 13 public transcriptome datasets. Three subsets of interferon-stimulated genes (ISGs) were selected for detailed examination, including one set of marker genes for type I IFN signaling (ISGa) and two sets of marker genes for type II IFN signaling (IFN-γ signaling, GBPs for the GBP gene cluster, and HLAd for the HLA-D gene cluster). In natural infection, activation of ISGa and GBPs was accompanied by the suppression of HLAd in hospitalized patients. Suppression of GBPs was also observed in certain critical conditions. The scale of regulation was much greater for ISGa than that of GBPs and HLAd. In addition, the suppression of HLAd was correlated with disease severity, and it took much longer for HLAd to return to the level of healthy controls than that for ISGa and GBPs. Upon viral challenge, the activation of ISGa and GBPs was similar to that of natural infection, while the suppression of HLAd was not observed. Moreover, GBPs’ return to the pre-infection level was at a faster pace than that of ISGa. Upon COVID-19 vaccination, activation was observed for all of these three gene sets, and the scale of activation was comparable for ISGa and GBPs. Notably, it took a much shorter time for GBPs and ISGa to return to the level of healthy controls than that in COVID-19 infection. In addition, the baseline values and transient activation of these gene sets were also associated with subsequent vaccination response. The intricate balance of IFN signaling was demonstrated in mild breakthrough infection, where attenuated response was observed in people with prior vaccination compared to that in vaccine-naïve subjects. Overall, distinctive temporal profiles of IFN signaling were observed in natural infection, viral challenge, and vaccination. The features observed in this work may provide novel insights into the disease management and vaccine development.

## 1. Introduction

Although the COVID-19 pandemic is in the rearview mirror, there are still many puzzling problems regarding the disease mechanism, including the roles of the host response. Interferon signaling is at the frontline of host defense against viral infection [1]. However, IFN signaling is highly complex, involving multiple pathways and hundreds of genes [2]. For example, type I IFN signaling pathway and type II IFN pathway (IFN-γ signaling) are two of the well-studied pathways in viral infections including COVID-19 [3,4]. In the past few years, diverse observations have been reported in the literature regarding the roles of these IFN signaling pathways in COVID-19.

In a study with a longitudinal sampling strategy [5], patients with mild COVID-19 who progressed to the severe stage had higher expression of marker genes for type I IFN signaling at baseline than non-progressors. In addition, cross-sectional comparison at baseline showed reduced activation of those marker genes in patients with severe COVID-19 compared to patients with mild COVID-19, which was attributed to the presence of IFN suppressors in multiple cell types. Thus, both activation and suppression of type I IFN signaling may be associated with severe COVID-19. In another study [6], the activation level of type I IFN signaling in the first 10 days did not show prognostic value on subsequent disease severity. These seemly contradictive observations demonstrate the complex relationship between disease severity and interferon signaling.

Complex regulation of IFN-γ signaling has also been reported. In a study of cytokines [7], a decreasing level of IFN-γ was observed from mild to moderate and severe groups of COVID-19 patients. In a study of genes encoding cytokines [8], *IFNG* expression was significantly reduced in COVID-19 patients compared to that in controls. In another study of both cytokines and gene expression in COVID-19 [9], an increased level of IFN-γ was observed in mild patients and outpatients but not in ICU patients compared to healthy controls. In addition, a decreasing level of IFN-γ-stimulated genes was observed from outpatients to mild patients to ICU patients. Thus, regulation of IFN-γ signaling may also be associated with severe COVID-19.

Another layer of complexity is the difference in IFN signaling with infection by different variants of the virus. Type I IFN response may be more robust in people infected with Omicron than those with pre-Omicron variants [10]. Decreased expression of certain ISGs in people infected with Omicron may be associated with poor outcome [11]. Higher expression of IFNs and ISGs in the nasopharynx may also be associated with good prognosis [12].

Studies on disease intervention have been reported using IFN-related strategies. In a rhesus macaque model, suppression of type I IFN signaling reduced viral replication and attenuated inflammation and pathogenesis [13]. This suggests that activation of type I IFN signaling could be detrimental to COVID-19 patients. In a case report [14], an ICU patient with protracted COVID-19 responded well to the IFN-γ treatment, accompanied by an increase in monocytic HLA-DR expression. This suggests that activation of IFN-γ signaling could be beneficial to COVID-19 patients. However, in meta-analysis of clinical trials, neither beneficial nor detrimental effect was observed for the treatment of COVID-19 using IFNs [15,16,17]. Altogether, complex roles of interferon signaling in COVID-19 have emerged.

In order to achieve better understanding of the roles of IFN signaling in COVID-19, it may be necessary to have a more in-depth examination of ISGs in COVID-19. In addition to the examination of natural infection, it may also be helpful to have a comparative examination in viral challenge and vaccination [18]. Since there is generally a delay, with an uncertain number of days between the actual infection and sampling in studies of natural infection, viral challenge experiments provide a more complete picture of the temporal profile (clinical course or evolvement of features over time) in mild infection. As for vaccination, the rationale is that host response to vaccination is unlikely harmful to the body for the vast majority of vaccinated people [19,20]. This is in contrast with the dual effect of host response in natural infection. Additionally, the comparative investigation may also provide novel insights for the development of better vaccines [21,22].

In this work, we selected three sets of marker genes for the examination of IFN signaling in natural infection, viral challenge, and vaccination. The gene selection was mainly based on an investigation of marker genes specific for type I IFN signaling and IFN-γ signaling [23]. In addition, findings from our previous works on blood transcriptome were also considered [24,25,26,27,28]. The selected marker genes included one gene set for type I IFN signaling and two gene sets for IFN-γ signaling. We examined the temporal profiles of these three sets of marker genes using public transcriptome datasets on natural infection, viral challenge, and vaccination. Distinctive patterns were observed under different scenarios. The implication of these observations to disease management as well as vaccine development is herein discussed.

## 2. Materials and Methods

The majority of the transcriptome datasets analyzed in this work were obtained by keyword searching (“COVID-19”) in the gene expression omnibus (GEO, https://www.ncbi.nlm.nih.gov/geo/, accessed on 5 June 2025). Other data sources were described in the specific datasets. The tissue sources for these studies were restricted to whole blood for consistency in comparison. The transcriptome experiments were mainly conducted on RNA-Seq (RNA sequencing) platforms, while only one dataset was found on a microarray platform. There were a total of thirteen datasets and 1827 samples. A summary of the datasets is presented in Table 1. More information can be found in Supplementary Table S1.

### 2.1. Transcriptome Datasets on Natural Infection

Two of the datasets were focused on the comparison between hospitalized COVID-19 patients and healthy controls. In the dataset GSE152641 [29], around 37% of the patients were intubated. The median time lapse between onset symptoms and sampling was 6 days (mostly between 4 and 8 days) for the COVID-19 patients. In another dataset (GSE243217), the comparison among COVID-19 sepsis, bacterial sepsis, and healthy controls was presented [30]. Around 85.7% and 77.2% of the subjects were intubated for patients with COVID-19 and bacterial sepsis, respectively. The blood samples were collected at hospital admission with unknown delay from onset symptoms.

COVID-19 patients with different severity levels and time information were examined in two datasets. In one dataset (https://data.mendeley.com/datasets/8wxhhykfnh/2/, accessed on 5 June 2025), flu patients were also included for comparison [31]. In this dataset, the mean time lapse between onset symptoms and sampling was 10.2 (2–30), 11.4 (1–19), 9.2 (1–21), 21.4 (6–37), and 7.3 (2–21) days for the OXY0, OXY1, TUBE-early, TUBE-late, and INFL groups, respectively. In the dataset Zenodo 6120249 (https://zenodo.org/records/6120249, accessed on 5 June 2025), sepsis patients were also included for comparison [32]. In this dataset, samples were collected at acute admission to the hospital. The time lapse between onset symptoms and sampling was 5–73, 1–37, and 0–58 days for the healthcare workers, hospitalized COVID-19 patients, and sepsis patients, respectively.

A longitudinal sampling strategy was adapted in two datasets. In the dataset GSE213313 [33], patients were sampled at three time points (enrollment, 1–4 days later, and 5–11 days later). There were 15 patients in the non-critical group and 19 patients in the critical group. At enrollment, the mean time lapse from onset symptoms was around 9.5 days for both groups. In another dataset (https://www.covid19cellatlas.org/patient/citiid/, accessed on 5 June 2025), patients were sampled at day 0 and day 28 after enrollment [34]. The patient groups included asymptomatic screening (A), symptomatic screening (B), hospitalized without oxygen requirement (C), hospitalized with supplementary oxygen (D), and critical (E). The mean time lapse between onset symptoms and baseline sampling was 6.5, 11.4, 10.6, and 24.6 for the B, C, D, and E groups, respectively. For clarity, the patients requiring ECMO support were redefined as group F in the data analysis.

### 2.2. Transcriptome Dataset on Viral Challenge

Besides natural infection, a dataset for SARS-CoV-2 challenge was also included (https://www.ebi.ac.uk/biostudies/arrayexpress/studies/E-MTAB-12993/, accessed on 5 June 2025) [35]. A total of 36 healthy and naïve young volunteers were intra-nasally inoculated with a pre-Alpha SARS-CoV-2 virus. Based on the PCR results, 17 subjects were defined as the infection group, and the other 19 subjects were defined as the non-infection group. The blood samples were collected at day −1, 0, 1, 2, 3, 4, 5, 7, 10, 14, and 28.

### 2.3. Transcriptome Datasets on Vaccination

The six transcriptome studies on COVID-19 vaccination employed different experimental designs. One study emphasized high-frequency sampling during the first two weeks of mRNA vaccination (GSE190001) [36]. Two other studies compared transcriptional change upon mRNA vaccination or mixed vaccination (GSE247401 and GSE199750) [37,38]. Another study focused on transcriptional change in high responders and low responders (GSE246525) [37]. One study examined response to mRNA vaccination in immunocompromised individuals (GSE250023) [39]. Another study investigated breakthrough infection with or without prior vaccination (GSE228839) [40].

### 2.4. Data Analysis of Transcriptional Response to Infection and Vaccination

Fifteen marker genes (three gene sets) were selected for the representation of IFN signaling. The expression values of the marker genes were extracted from the transcriptome datasets described above. The raw gene counts were normalized and log2 transformed for the downstream analyses. The mean expression of each of the three gene sets was calculated for every sample. The samples in each dataset were grouped according to the original experimental design. The median expression values of the marker gene sets from the control group were used for data normalization, where every data point was subtracted by these reference values. The statistical calculations were carried out in R (https://www.r-project.org/, accessed on 5 June 2025). The figures were drawn using the ggplot2 package in R. The two group comparisons were performed using *t*-test. The multi-group comparisons were performed using one-way ANOVA followed by Tukey test (Tukey honest significant differences).

## 3. Results

### 3.1. Selection of Marker Genes for IFN Signaling

As described earlier, the selection of marker genes for IFN signaling was mainly based on a previous experimental work on marker genes specific for type I IFN signaling and IFN-γ signaling [23]. We selected five genes to represent type I IFN signaling (gene set ISGa) and ten genes for IFN-γ signaling (gene sets GBPs and HLAd). The gene set ISGa included *IFI27*, *IFI44L*, *ISG15*, *RSAD2*, and *IFITM3*. The gene set GBPs included *GBP1*, *GBP2*, *GBP3*, *GBP4*, and *GBP5*. The gene set HLAd included *HLA-DRA*, *HLA-DRB1*, *HLA-DMA*, *HLA-DMB*, and *HLA-DPA1*. When selecting genes for the ISGa gene set, we first considered the top ten ISGs from our previous analysis of infectious diseases and immune disorders [41]. Among these genes, *IFI27*, *IFI44L*, and *ISG15* were in the short list of type I IFN-specific genes from the above-mentioned experiment [23]. In addition, *RSAD2* and *IFITM3* were among the consensus up-regulated genes in our previous meta-analysis of COVID-19 transcriptome datasets [27]. For the HLAd gene set, these five genes were all in the short list of IFN-γ-specific genes from the experiment. As for the GBPs gene set, *GBP1*, *GBP2*, and *GBP5* were in the short list of IFN-γ-specific genes from the experiment. Two other genes, *GBP3* and *GBP4*, in the same gene cluster were added to the gene set because these genes are within a small chromosomal region and likely co-regulated.

For each gene set, the mean expression values of the five marker genes were used for the data presentation. Additionally, the median values of ISGa, GBPs, and HLAd in the control group were used for data normalization to reflect the deviation from the reference point. A total of thirteen transcriptome datasets were examined to reflect various aspects of natural infection, viral challenge, and vaccination (Table 1 and Table S1).

### 3.2. IFN Signaling in Hospitalized Patients with COVID-19

**Figure 1:**
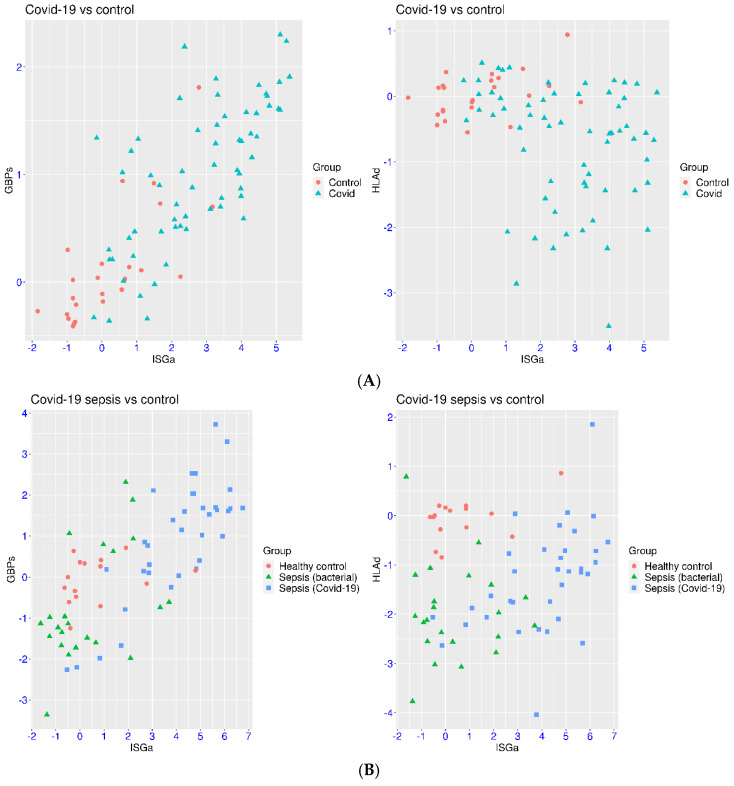
Regulation of ISGs in patients with COVID-19. (**A**) Upper panel: Comparison of IFN signaling in hospitalized COVID-19 patients and that in healthy controls (GSE152641) using 2D maps of three marker gene sets (ISGa, GBPs, and HLAd). (**B**) Lower panel: Comparison of IFN signaling in COVID-19 sepsis with that in bacterial sepsis and healthy controls (GSE243217) using 2D maps of three marker gene sets (ISGa, GBPs, and HLAd).

First, hospitalized patients with COVID-19 were compared with healthy controls in a study involving the dataset GSE152641 (Figure 1A). It was evident that both ISGa and GBPs were activated, while HLAd was suppressed in COVID-19 patients compared to healthy controls. For the healthy controls, the expression values were mostly within [−2, 3] for ISGa and [−1, 1] for both GBPs and HLAd. There was only one exception for ISGa and GBPs each and no exceptions for HLAd. Among the 62 patients, 31 patients (50.0%) had ISGs > 3, 33 patients (53.2%) had GBPs > 1, and 20 patients (32.2%) had HLAd < −1. The *p*-values were 8.21 × 10^−10^, 6.38 × 10^−8^, and 8.34 × 10^−7^ for the comparison of ISGa, GBPs, and HLAd in the two sample groups, respectively. Within this cohort, relatively strong correlation was observed between ISGa and GBPs (r = 0.81). However, much higher activation was observed for ISGa than for GBPs. The median expression values in the patient group were 2.93, 1.03, and −0.47 for ISGa, GBPs, and HLAd, respectively. Additionally, high heterogeneity was observed for all three gene sets in the patient group. This was likely due to the combination of factors such as disease severity (37% intubated), sampling time (mostly 4–8 days from onset symptoms), and pre-infection status of IFN signaling (unknown) within the patient group.

In another dataset (GSE243217), a severe form of COVID-19 (sepsis) was examined. Some consistent features were observed (Figure 1B), including the suppression of HLAd and greater activation of ISGa than that of GBPs. The control samples also had similar expression of ISGa, GBPs, and HLAd compared to the previous dataset. The median expression values in the COVID-19 sepsis group were 4.66, 1.15, and −1.15 for ISGa, GBPs, and HLAd, respectively. The *p*-values were 1.15 × 10^−7^, 1.23 × 10^−3^, and 2.93 × 10^−7^ for the comparison of ISGa, GBPs, and HLAd in the two sample groups, respectively. Compared to the previous dataset, some notable differences were observed in COVID-19 sepsis. For example, the scale of activation for ISGa and GBPs was higher in some of the patients with COVID-19 sepsis (ISGa > 6 or GBPs > 3) than that in other hospitalized COVID-19 patients. Suppression of HLAd was observed in a higher proportion of patients with COVID-19 sepsis (HLAd < −1 in 62.9% of the patients) than in hospitalized patients. Unexpectedly, suppression of GBPs (GBPs < −1) was observed in four patients with COVID-19 sepsis, and activation of HLAd (HLAd > 1) was observed in one patient with COVID-19 sepsis. In the same dataset, suppression of HLAd and GBPs was observed in the majority of patients with bacterial sepsis (90.5% and 52.4%). In the meantime, activation of GBPs or ISGa was also observed in a few patients with bacterial sepsis (three and two). Thus, sepsis condition may induce additional regulation of these marker gene sets in both directions. This should be taken into account when understanding the gene regulation in COVID-19 sepsis.

### 3.3. Disease Severity, Time, and IFN Signaling in Natural Infection

As described earlier, IFN signaling may vary in COVID-19 patients with different severity levels. The information on disease severity was available in a relevant dataset (Mendeley 8wxhhykfnh.2). The COVID-19 samples were divided into four groups, including the OXY0 group for patients without the requirement of supplementary oxygen, the OXY1 group for patients requiring supplementary oxygen, and the TUBE_early and TUBE_late groups for patients requiring intubation (early and late stages).

First, the comparison between male and female patients or the younger and older patients (age > 60) did not reach statistical significance for any of the three gene sets (*p* > 0.3 for the sex comparison and *p* > 0.8 for the age comparison, Supplementary Figure S1). Thus, age and sex may not have significant impact on IFN signaling upon viral infection.

**Figure 2:**
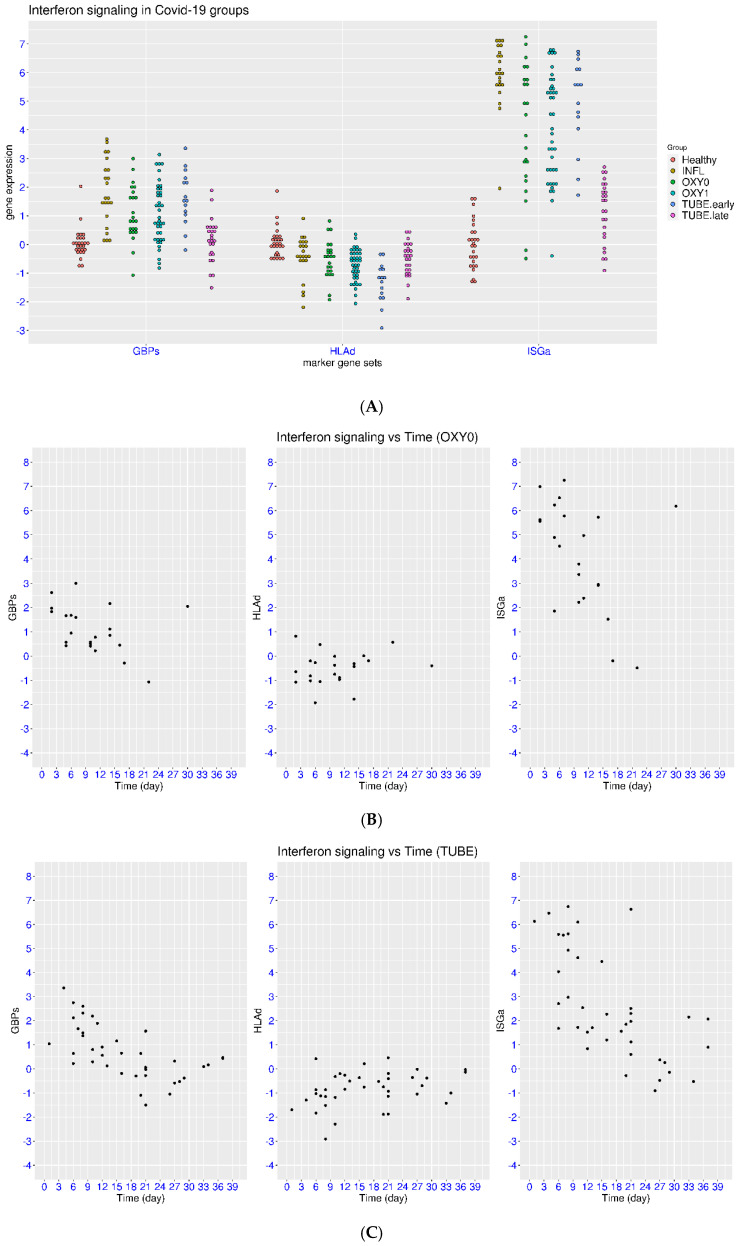
Regulation of ISGs in COVID-19 patients at different time and severity levels (dataset Mendeley 8wxhhykfnh.2). (**A**) Upper panel: Comparison of IFN signaling in COVID-19 patients with that in flu patients and healthy controls using three marker gene sets (ISGa, GBPs, and HLAd). Four groups of COVID-19 patients were recruited, including hospitalized patients with or without the requirement of supplementary oxygen or intubation. (**B**) Middle panel: Regulation of IFN signaling at different time (days from onset symptoms) in the OXY0 group (no oxygen requirement). (**C**) Lower panel: Regulation of IFN signaling at different time (days from onset symptoms) in the combined TUBE group (intubated patients).

A significant portion of COVID-19 patients had activation of ISGa or GBPs and suppression of HLAd compared to the control group (52.5%, 42.6%, and 32.7%, Figure 2A). Notably, an increasing level of suppression for HLAd was observed from the OXY0 group (median value −0.43) to the OXY1 group (median value −0.66) and the TUBE_early group (median value −1.19). The *p*-values were 9.93 × 10^−4^ and 0.015 for the comparison of HLAd in the TUBE_early group with that in the OXY0 or OXY1 group, respectively. In contrast, the *p*-values were >0.36 for the comparison of either ISGa or GPBs among these three groups. This indicates that only HLAd was correlated with disease severity. It was also evident that the expression of the marker genes was much closer to the level of healthy controls in the TUBE_late group compared to that in the TUBE_early group. The *p*-values for the comparison between the TUBE_early group and TUBE_late group were <7.27 × 10^−4^ for the three gene sets. Meanwhile, the comparison between the TUBE_late group and control group reached statistical significance only for HLAd (*p* = 0.011), indicating a slower pace for HLAd to return to the level of healthy controls than that for GBPs and ISGa.

The information on time was available for this dataset, making it possible to further examine the dynamics of IFN signaling. For the OXY0 group, a fast return to the level of healthy controls was observed for these marker genes (Figure 2B). Among the ten samples in week one, nine samples had ISGa > 3 (90%), and seven samples had GBPs > 1 (70%). This decreased to four samples with ISGa > 3 (50%) and two samples with GBPs > 1 (25%) among the eight samples in week two, indicating a faster pace for the return to the level of healthy controls for GBPs than that for ISGa. Among the four samples taken at a later time, only one sample had ISGa > 3 and GBPs > 1 at day 30, which could be due to a new viral infection. As for the suppression of HLAd (<−1), there were four samples in week one (40%), one sample in week two (12.5%), and none at the later time. Overall, the vast majority of patients with mild COVID-19 had expression of GBPs and HLAd that returned to the level of healthy controls in week two, while it generally took a few more days for ISGa to return to the level of healthy controls.

A slower pace of return to the level of healthy controls for the marker genes was observed in patients with more severe disease than in the mild patient group. For the OXY1 group (Supplementary Figure S2A), there were twelve, ten, and five patients with regulation of ISGa, GBPs, and HLAd among the twelve patients in week one (100%, 83.3%, and 41.7%). This changed to nine, six, and six for the regulation of ISGa, GBPs, and HLAd among the eighteen patients in week two (50%, 33.3%, and 33.3%). In week three, there were still six, three, and one patients among the ten samples with the regulation of ISGa, GBPs, and HLAd (60%, 30%, and 10%), respectively.

For the combined TUBE group, a slower pace was also observed for the marker genes to return to the level of healthy controls than in the mild patient group (Figure 2C). Using the same cutoff, five of the seven patients had activation of ISGa or GBPs or suppression of HLAd (71.4% each) in week one. This changed to five, six, and five patients for the regulation of ISGa, GBPs, and HLAd among the eleven patients in week two (45.5%, 54.5%, and 45.5%). In week three, there were two, three, and three patients among the twelve samples with regulation of ISGa, GBPs, and HLAd (16.7%, 25%, and 25%), respectively. Moreover, three of the patients had HLAd < −1 even after week three. Overall, it may take a longer time for the marker genes to return to the level of healthy controls in patients with severe or critical COVID-19 than in patients with mild COVID-19, especially for HLAd.

In this dataset, the comparison with mild flu patients (INFL group) was also available (Supplementary Figure S2B). There were sixteen, twelve, and four patients with the regulation of ISGa, GBPs, and HLAd among the seventeen samples taken in week one (94.1%, 70.6%, and 23.5%). The proportion of activation for ISGa and GBPs was similar to that in week one of the OXY0 group, while a lower proportion of the INFL group had suppression of HLAd than did the OXY0 group. Further comparison was not possible because only three samples were taken after week one for the INFL group.

**Figure 3:**
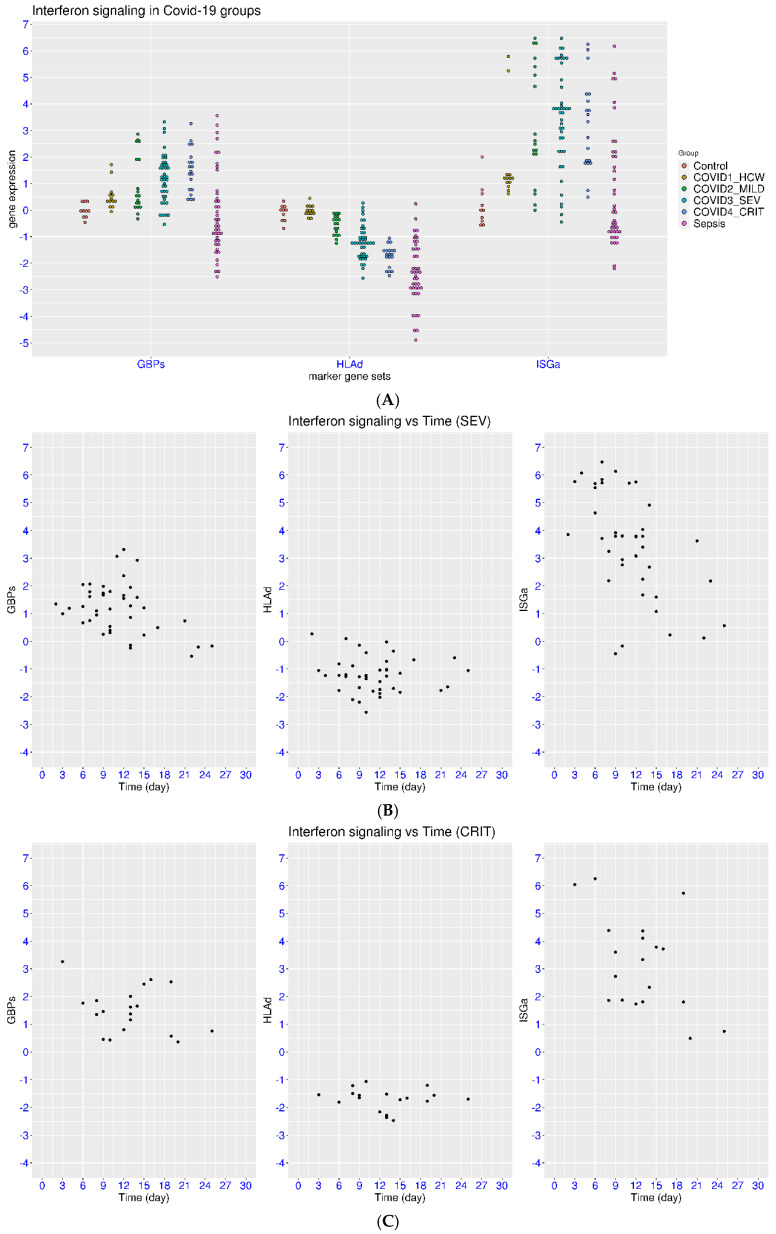
Regulation of ISGs in COVID-19 patients at different time and severity levels (dataset Zenodo 6120249). (**A**) Upper panel: Comparison of IFN signaling in COVID-19 patients with that in sepsis patients and healthy controls using three marker gene sets (ISGa, GBPs, and HLAd). Four groups of COVID-19 patients were recruited, including recovering healthcare workers and hospitalized patients (mild, severe, and critical). (**B**) Middle panel: Regulation of IFN signaling at different time (days from onset symptoms) in the severe COVID-19 group (supplementary oxygen). (**C**) Lower panel: Regulation of IFN signaling at different time (days from onset symptoms) in the critical COVID-19 group (intubated patients).

Similar features were observed in another dataset (Zenodo 6120249). Consistently, a significant portion of patients with COVID-19 had activation of ISGa or GBPs and suppression of HLAd (50.5%, 50.5%, and 56%, Figure 3A). Notably, suppression of HLAd was not observed in the group of healthcare workers who were tested positive but not admitted to the hospital (median value −0.06, *p* = 1 when compared with controls). For the three groups of hospitalized patients with COVID-19, an increasing level of suppression of HLAd was observed from the mild group (median value −0.51) to the severe group (median value −1.23) and critical group (median value −1.66). The *p*-values all reached statistical significance for the comparisons among the patient groups. This trend again demonstrated a strong correlation between disease severity and suppression of HLAd. This dataset also included samples with all-cause sepsis for comparison. It was clear that sepsis may induce even greater suppression of HLAd (median value −2.35) than COVID-19. In the meantime, sepsis may also induce both activation and suppression of ISGa and GBPs, consistent with the observation in Figure 1B.

More details were revealed when the time information was included. For the healthcare worker group (Supplementary Figure S3A), only two patients had activation of ISGa and GBPs (day 7 and day 8), and no suppression of HLAd was observed. For the patients in the mild COVID-19 group (Supplementary Figure S3B), only five, four, and one patient had regulation of ISGa, GBPs, and HLAd among the eleven patients in week one (45.5%, 36.4%, and 9.1%). This changed to two, two, and one patients with the regulation of ISGa, GBPs, and HLAd among the five patients in week two (40%, 40%, and 20%). No regulation of the marker genes was observed for the two patients after week two. Consistent with the previous dataset, regulation of IFN signaling was mainly observed within the first two weeks for patients with mild COVID-19.

For the patients with severe COVID-19 (Figure 3B), ten, eight, and seven patients had regulation of ISGa, GBPs, and HLAd among the ten patients in week one (100%, 80%, and 70%). This changed to sixteen, sixteen, and eighteen patients with the regulation of ISGa, GBPs, and HLAd among the twenty-four patients in week two (66.7%, 66.7%, and 75%). This further changed to one, one, and three patients with the regulation of ISGa, GBPs, and HLAd among the four patients in week three (25%, 25%, and 75%). This was followed by the suppression of HLAd in two of three patients in week four (66.7%). This again demonstrated the slower pace for the marker genes, especially HLAd, to return to the level of healthy controls in patients with severe COVID-19 than that in patients with mild COVID-19.

For the patients with critical COVID-19 (Figure 3C), the two patients in week one both had regulation of all three gene sets (100% each). This changed to five, eight, and eleven patients with the regulation of ISGa, GBPs, and HLAd among the eleven patients in week two (45.5%, 72.7%, and 100%). This further changed to three, three, and five patients with the regulation of ISGa, GBPs, and HLAd among the five patients in week three (60%, 60%, and 100%). This was followed by the suppression of HLAd in a sole patient in week four (100%). Overall, the marker genes, especially HLAd, showed a much slower return to the level of healthy controls in patients with critical COVID-19 compared to that in other patient groups.

**Figure 4:**
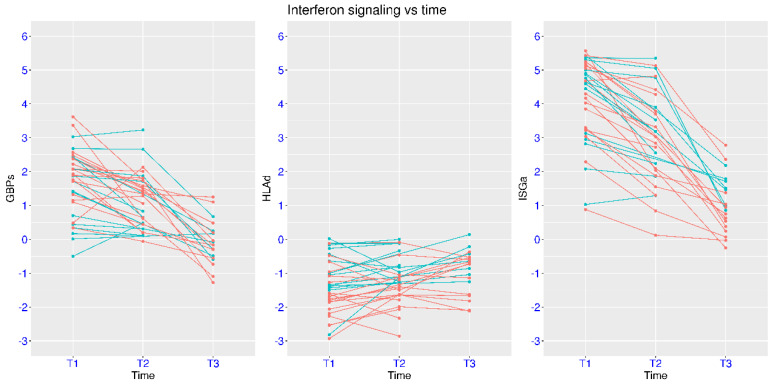
Regulation of ISGs in COVID-19 patients at two severity levels and three time points (GSE213313). Temporal profiles of IFN signaling in COVID-19 patients were plotted using three marker gene sets (ISGa, GBPs, and HLAd). Two groups of COVID-19 patients (cyan for non-critical and red for critical) were monitored at three time points. T1, baseline at hospital admission; T2, 1–4 days after enrollment; T3, 5–11 days after enrollment.

The dynamics of IFN signaling were also examined in the dataset GSE213313 with longitudinal sampling. Two groups of COVID-19 patients (critical and non-critical) were monitored at three time points (enrollment, 1–4 days, and 5–11 days later). Again, activation of ISGa and GBPs and suppression of HLAd were observed at enrollment for both groups (median 9.5 days from onset symptoms, Figure 4). In addition, the critical group had significantly lower HLAd than the non-critical group (*p* = 0.035). During the disease course, the return to the level of healthy controls for these three gene sets occurred at difference paces. From T1 to T3, ISGa significantly decreased from 4.64 to 1.51 (median values) for the non-critical group and from 4.30 to 0.63 for the critical group. During the same process, GBPs decreased from 1.42 to −0.08 (median values) for the non-critical group and from 2.07 to −0.17 for the critical group. In the meantime, HLAd increased from −1.01 to −0.65 (median values) for the non-critical group and from −1.76 to −0.73 for the critical group. At T3, only HLAd of the critical group reached statistical significance (*p* = 3.61 × 10^−4^) when compared with the control group. Overall, it was the fastest for GBPs and slowest for HLAd to return to the level of healthy controls in patients with severe and critical COVID-19.

**Figure 5:**
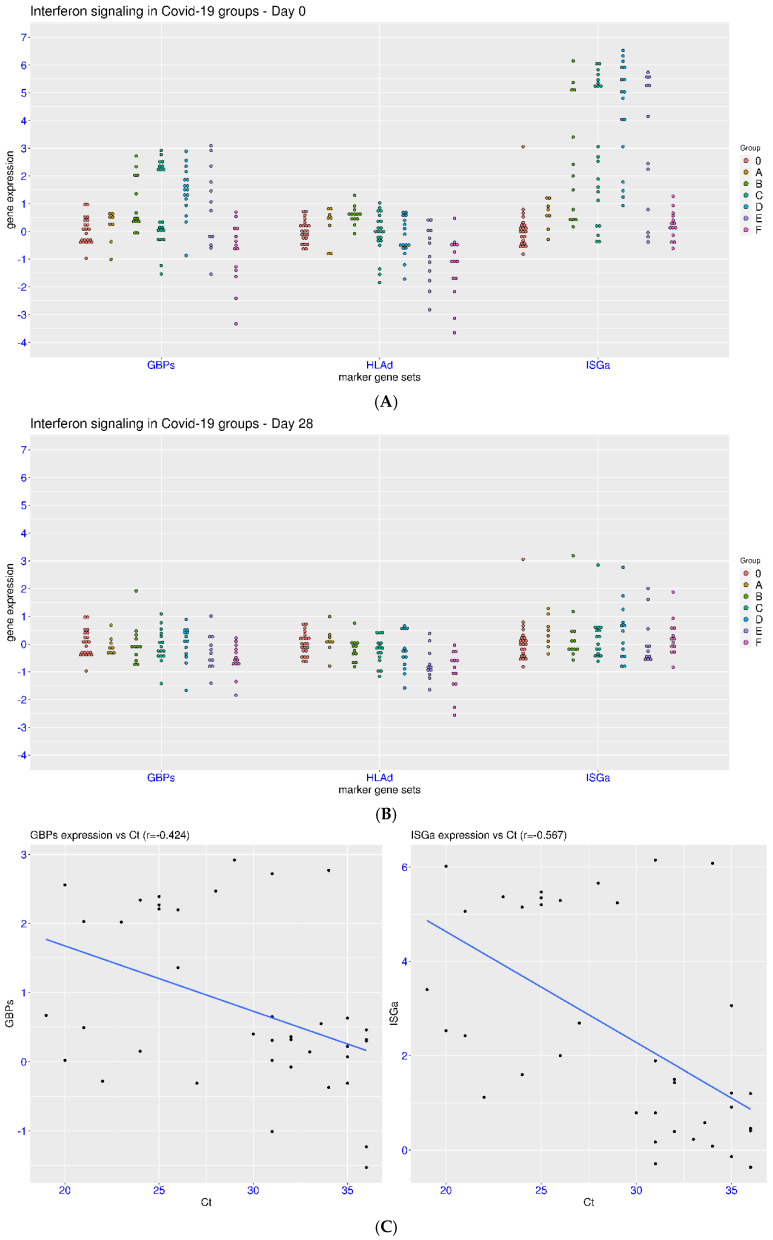
Regulation of ISGs in COVID-19 patients at different severity levels and two time points (COVID-19 cell atlas). Dynamic regulation of IFN signaling in COVID-19 patients was plotted using three marker gene sets (ISGa, GBPs, and HLAd). Six groups of COVID-19 patients were monitored at two time points after enrollment ((**A**) upper panel, day 0; (**B**) middle panel, day 28). (**C**) Lower panel: Correlation between GBPs/ISGa and Ct at enrollment plotted for patients with mild infection (A, B, and C groups). The blue lines indicate linear fitting of the data. Group IDs: 0, control; A, asymptomatic; B, symptomatic at screening; C, hospitalized and no oxygen required; D, supplementary oxygen required; E, intubated; F, ECMO support required.

In another dataset (COVID-19 cell atlas), COVID-19 patients with different severity levels were monitored at two time points (day 0 and 28 after enrollment). There were six groups of patients, including asymptomatic (group A), symptomatic and non-hospitalized (group B), hospitalized without supplementary oxygen (group C), hospitalized with supplementary oxygen (group D), intubated patients (group E), and patients with ECMO support (group F). At day 0, no regulation of ISGa, GBPs, and HLAd (ISGa > 3 or GBPs > 1 or HLAd < −1) was observed for the asymptomatic patients (Figure 5A). In contrast, activation of ISGa and GBPs was observed in patient groups B, C, D, and E. In group F, no activation of ISGa or GBPs was observed. Rather, suppression of GBPs (GBPs < −1) was observed in five of the fourteen patients in group F, similar to the feature in sepsis. Suppression of HLAd (HLAd < −1) was observed in some of the hospitalized patients, and a clear trend was observed between HLAd expression and disease severity. The median values of HLAd were 0.00, −0.47, −0.66, and −1.07 for patients in the C, D, E, and F groups, respectively. Statistical significance was reached when the HLAd in the E or F group was compared with that in other groups (except for the D/E comparison). In contrast, an upward shift for HLAd (median value 0.64) was observed in group B. This was an interesting observation for symptomatic patients without the need for hospital admission. Thus, more heterogeneity of gene regulation was observed with more precise partition of patients.

At day 28, a return to the level of healthy controls for ISGa and GBPs was observed for the vast majority of patients (all but one for ISGa and three for GBPs, Figure 5B). However, suppression of HLAd (HLAd < −1) was still observed for some of the hospitalized patients. There were two, two, three, and six patients with the suppression of HLAd in the patient groups C, D, E, and F, respectively. This again demonstrated that the return to the level of healthy controls for HLAd was the slowest among the three gene sets.

Additionally, the Ct values of PCR tests at enrollment were provided in this dataset. It was interesting that a weak to moderate correlation was observed between the Ct values and GBPs expression or ISGa expression for patients in groups A, B, and C (asymptomatic or mild COVID-19) (Figure 5C). From the Pearson test, the r values were −0.567, −0.425, and 0.019 for ISGa, GBPs, and HLAd, respectively. From the Spearman test, the rho values were −0.598, −0.410, and 0.034 for ISGa, GBPs, and HLAd, respectively. Activation of ISGa or GBPs was rarely observed in patients with large Ct value (30 and above). This might suggest that the activation of ISGa and GBPs is partially attributed to the viral load. However, this correlation was not as obvious when the patients in the other groups were included. A confounding factor was the time lapse from onset symptoms to sampling, which varied among the patient groups (much longer for patients in the groups E and F). No correlation was observed between the Ct values and HLAd expression.

### 3.4. Regulation of IFN Signaling upon SARS-CoV-2 Challenge

**Figure 6:**
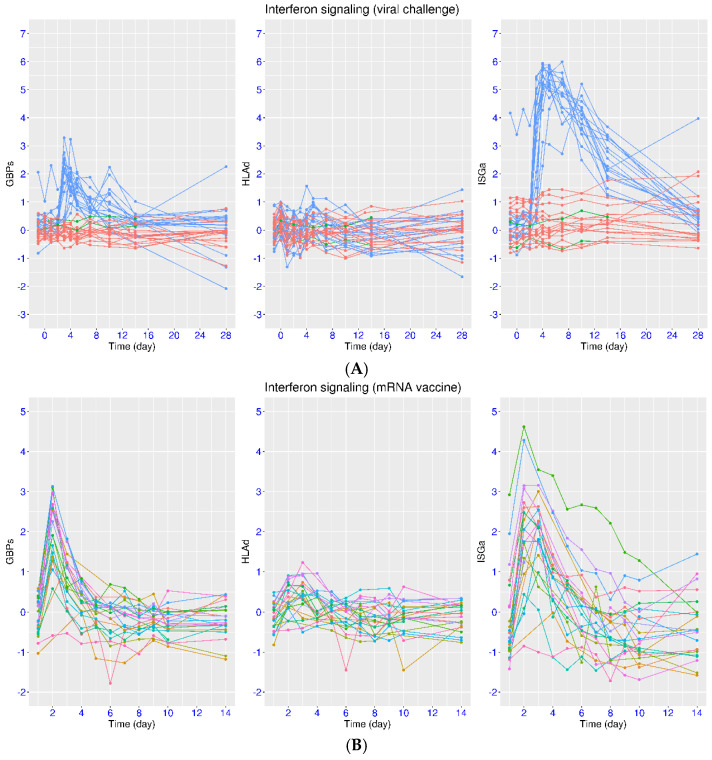
Regulation of ISGs in volunteers upon SARS-CoV-2 challenge or mRNA vaccination. (**A**) Upper panel: Temporal profiles of IFN signaling after SARS-CoV-2 challenge (E-MTAB-12993) were plotted using three marker gene sets (ISGa, GBPs, and HLAd). Three groups of subjects were monitored for 28 days (blue for infected, red for non-infected, and green for non-infected and seropositive). (**B**) Lower panel: Temporal profiles of IFN signaling after mRNA vaccination (GSE190001) were plotted using three marker gene sets (ISGa, GBPs, and HLAd). The volunteers were monitored for 14 days. Different colors indicate different subjects.

The variation in time lapse between onset symptoms and sampling is a major confounding factor when examining the regulation of IFN signaling in COVID-19 and other relevant diseases. Even when the time information was provided, it may not be accurate due to various reasons. This issue can be resolved in experiments with SARS-CoV-2 challenge. In one such study (dataset E-MTAB-12993), the volunteers were divided into two groups (infected or uninfected) based on the PCR results after the viral challenge. It was clear that activation of both ISGa and GBPs was observed in the infected group but not in the uninfected group (Figure 6A). Again, greater activation of ISGa than that of GBPs was observed according to the peak values, consistent with the feature in natural infection (Figure 1, Figure 2, Figure 3, Figure 4 and Figure 5). The median expression values of ISGa were 4.01, 5.25, 5.19, 4.87, 4.09, 2.28, and 0.48 on days 3, 4, 5, 7, 10, 14, and 28, respectively. The median expression values of GBPs were 2.03, 1.73, 1.42, 0.82, 0.69, 0.30, and 0.35 on days 3, 4, 5, 7, 10, 14, and 28, respectively. The activation of both gene sets started on day 3, but the process varied for the return to the level of healthy controls. For GBPs, the activation peaked on day 3 and was substantially reduced on day 7 (from 2.03 to 0.82). On the other hand, the activation of ISGa peaked on day 4 and lasted much longer, up to day 10 (from 5.25 to 4.09) and beyond. As for HLAd, only minor fluctuation was observed. The median expression values for HLAd were 0.33, 0.38, and 0.15 on days 4, 5, and 7, respectively. This was consistent with the observation from non-hospitalized symptomatic patients (Figure 5A).

It should be noted that the symptoms started around day 4 for the infected group. Thus, the time between day 4 to 10 in this dataset may correspond to the first week of natural infection (mild cases). According to this dataset, the activation of GBPs could be substantially reduced by the end of the first week in natural infection, while the activation of ISGa could be substantially reduced by the end of the second week in natural infection. Overall, a faster return to the level of healthy controls for GBPs than that for ISGa was consistently observed in both natural infection and viral challenge. Additionally, positive PCR results started earlier and were present on day 2.5 for the vast majority of volunteers in the infected group, and the infection lasted for about two weeks in this cohort, which corresponded better with the temporal profile of ISGa. It should be noted that this group of infected volunteers could be categorized into the mild infection group when compared with datasets on natural infection. It may not fully reflect features in the severe and critical groups.

### 3.5. Regulation of IFN Signaling upon Vaccination

Vaccination provides another opportunity to study host response, including IFN signaling. In the dataset GSE190001, the high-frequency sampling enabled the observation of fine-grained dynamic features in the early phase of vaccination. In contrast to natural infection or viral challenge, transient activation of all three gene sets was observed upon mRNA vaccination (Figure 6B). There was an increase of 2.69 (median value) for ISGa expression and an increase of 2.49 (median value) for GBPs expression from day 1 (baseline prior to vaccination) to day 2 (one day after vaccination). The activation of ISGa and GBPs was at a comparable scale after vaccination (2.69 vs. 2.49), which was also different from the feature observed after infection. The activation was sustained at day 3 for ISGa (median value 2.75), but it was significantly attenuated for GBPs (median value 1.21). The expression of both gene sets gradually came down to the baseline level in the next few days (0.43 at day 4 for GBPs and 0.58 at day 6 for ISGa). Again, the return to the baseline level was at a faster pace for GBPs than for ISGa. As for HLAd, the scale of activation was much smaller than that of ISGa and GBPs upon vaccination. The moderate activation started on day 2 (median value 0.37), peaked on day 3 (median value 0.52), and attenuated on day 4 (median value 0.13). This was similar to the observation in the viral challenge experiment.

**Figure 7:**
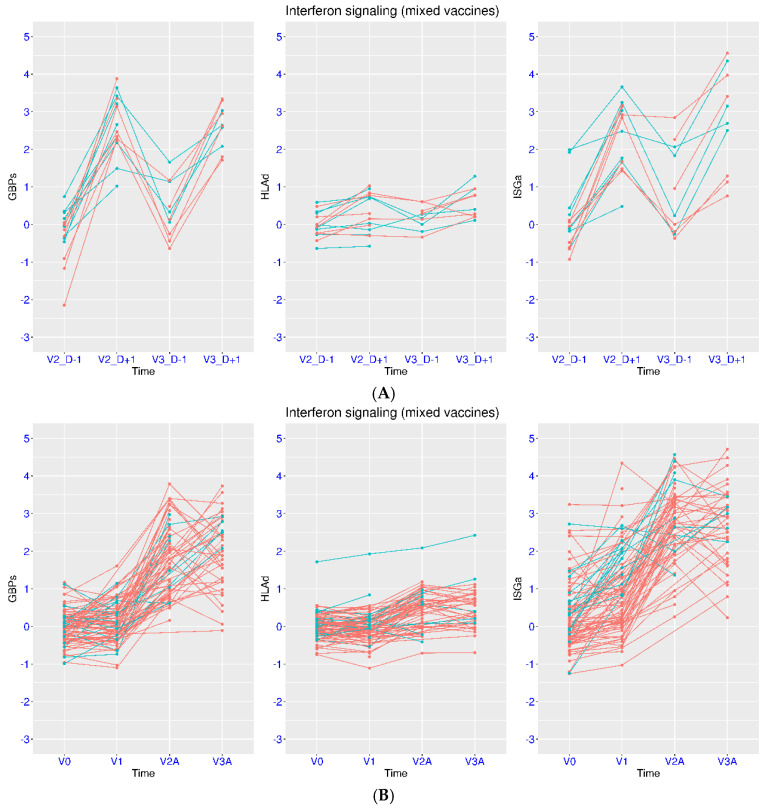
Regulation of ISGs in volunteers upon heterologous or homologous vaccination. (**A**) Upper panel: Temporal profiles of IFN signaling after vaccination (GSE247401) were plotted using three marker gene sets (ISGa, GBPs, and HLAd). Two vaccination schemes were included (cyan for mixed vaccines and red for mRNA vaccines). The subjects were monitored at four time points (one day before and after the second and third doses). (**B**) Lower panel: Temporal profiles of IFN signaling after vaccination (GSE199750) were plotted using three marker gene sets (ISGa, GBPs, and HLAd). Two vaccination schemes were included (cyan for mixed vaccines and red for mRNA vaccines). The subjects were monitored at four time points (baseline, seven days after the first dose, and one day after the second and third doses).

Consistent with the previous dataset, a similar feature was observed after heterologous vaccination. In the dataset GSE247401, one group chose mRNA vaccine as the primary dose, while the other group chose ChAdOx1 vaccine as the primary dose. Both groups chose mRNA vaccine as the second and third doses. Due to the small sample size, these two groups were combined in the analysis (Figure 7A). After the second dose, there was an increase of 2.56, 2.61, and 0.36 for the median expression of ISGa, GBPs, and HLAd, respectively. After the third dose, there was an increase of 2.33, 2.40, and 0.37 for the median expression of ISGa, GBPs, and HLAd, respectively. Statistical significance was reached for ISGa and GBPs after both doses.

A different vaccination scheme was employed in another dataset (GSE199750). One group used mRNA vaccine throughout the study, while the other group used ChAdOx1vaccine for the first and second doses and mRNA vaccine for the third dose (Figure 7B). Again, the overall transcriptional response in the mRNA vaccine group was similar to the observation from the previous dataset. After the second dose, there was an increase of 2.89, 1.81, and 0.61 from the baseline for the median expression of ISGa, GBPs, and HLAd, respectively. After the third dose, there was an increase of 2.92, 1.90, and 0.58 from the baseline for the median expression of ISGa, GBPs, and HLAd, respectively. The comparison between the baseline and either dose reached statistical significance for all three gene sets. In addition, an increase of 0.54 was observed for the median expression of ISGa from baseline to seven days after the first dose (*p* = 0.026), indicating a slower return to the baseline level for ISGa compared to GPBs and HLAd.

For the second group with heterologous vaccination, similar features were also observed. After the second dose, there was an increase of 2.98, 1.83, and 0.68 from the baseline for the median expression of ISGa, GBPs, and HLAd, respectively. After the third dose, there was an increase of 2.59, 2.49, and 0.36 from the baseline for the median expression of ISGa, GBPs, and HLAd, respectively. In addition, an increase of 1.55 was observed for the median expression of ISGa from baseline to seven days after the first dose (*p* = 0.0044), again indicating a slower return to the baseline level for ISGa compared to GPBs and HLAd.

### 3.6. Differential Host Transcriptional Response upon Vaccination

**Figure 8:**
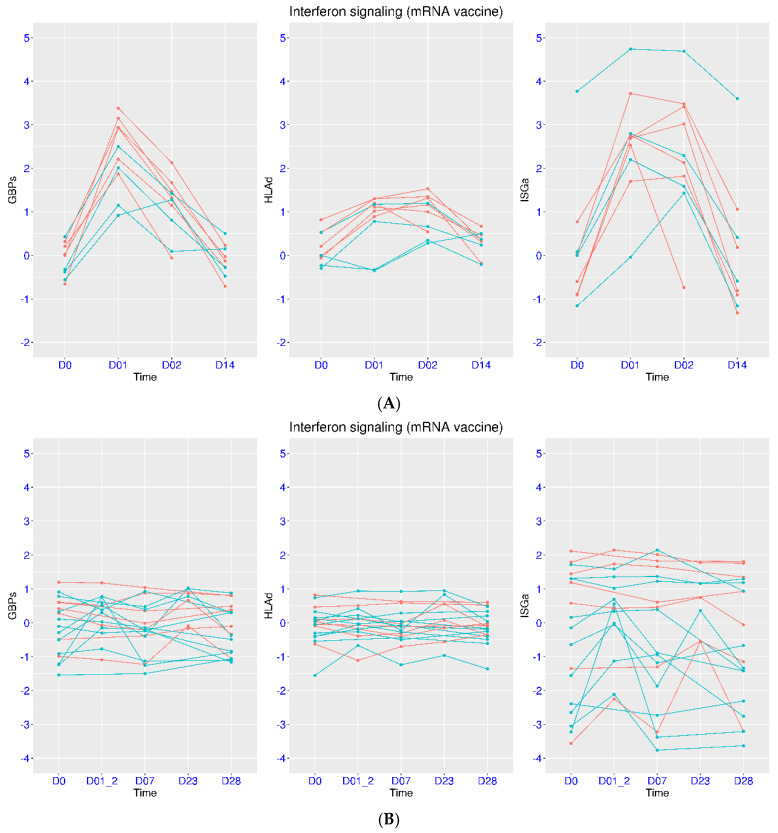
Regulation of ISGs in people with different response to vaccination. (**A**) Upper panel: Temporal profiles of IFN signaling after mRNA vaccination (GSE246525) were plotted using three marker gene sets (ISGa, GBPs, and HLAd). Two response groups were included (cyan for low response and red for high response). The healthy subjects were monitored at four time points (baseline and one, two, and fourteen days after the third dose). (**B**) Lower panel: Temporal profiles of IFN signaling after vaccination (GSE250023) were plotted using three marker gene sets (ISGa, GBPs, and HLAd). Two response groups were included (cyan for responder and red for non-responder), The SLE patients were monitored at five time points (baseline and one/two and seven days after the first dose and two and seven days after the second dose at day 21).

Upon vaccination, people may respond differently, which could be reflected on the immune gene expression. In the dataset GSE246525, the transcriptional response within the first two weeks of the third vaccination dose was examined for those who had high or low response to previous vaccinations (Figure 8A). These two groups were identified by assessing immune response from two tests (interferon gamma release assay and surrogate virus neutralization test). Among the four low responders, one had abnormally high ISGa at baseline (3.77). This subject also had a decrease in HLAd (−0.35) from day 0 to day 1. Another low responder also had a decrease in HLAd (−0.10) from day 0 to day 1. Among the six high responders, four subjects had the highest GBPs (>2.93) at day 1. In addition, six of the seven subjects with HLAd > 0.89 at day 1 were high responders. Thus, high responders and low responders had distinctive expression profiles of these three gene sets at baseline and day 1, indicating the prognostic value of these gene sets.

Vaccination is generally less efficacious for immunocompromised people, which could be reflected on the transcriptional response. In the dataset GSE250023, SLE patients were monitored after mRNA vaccination and were subsequently divided into responder and non-responder groups. Compared to the healthy subjects in Figure 8A, transcriptional activation was not observed for most of the SLE patients (Figure 8B). For the non-responders, transcriptional activation for GBPs and HLAd was not observed in any of the SLE patients at day 1/2. For the responders, transcriptional activation for ISGa, GBPs, and HLAd was only observed in about half of the SLE patients at day 1/2. Thus, the relative response to vaccination in SLE patients could also be reflected in the activation of these three gene sets.

### 3.7. Modulation of IFN Signaling in Mild Breakthrough Infection

**Figure 9:**
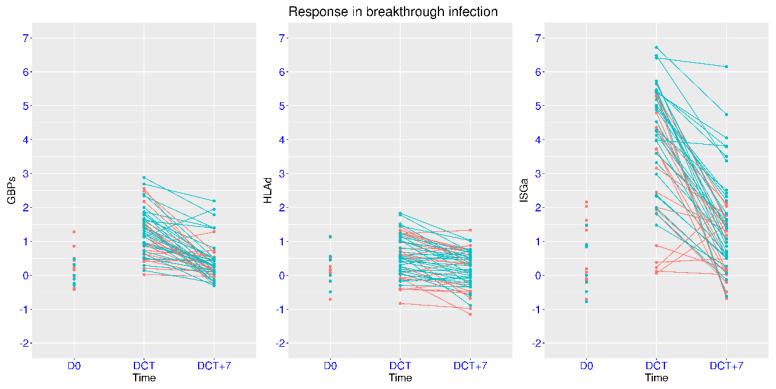
Regulation of ISGs in people with mild breakthrough infection. Temporal profiles of IFN signaling after breakthrough infection (GSE228839) were plotted using three marker gene sets (ISGa, GBPs, and HLAd). Two groups were included (cyan for people without prior vaccination and red for people with prior vaccination). The subjects were monitored at two time points (the day with first positive PCR and seven days later). Independent samples before infection were also included as reference. The two-group comparison for ISGa at the first time point (DCT) reached statistical significance (*p* = 0.0077).

Vaccination can provide immune protection for future infection, and the mechanism of protection has been examined in many studies. In the dataset GSE228839, people with or without prior ChAdOx1 vaccination were enrolled upon symptomatic infection (confirmed by PCR tests). The overall pattern of gene expression change was similar in these two groups, but notable difference was observed (Figure 9). For the unvaccinated group, there was an increase of 4.91, 1.36, and 0.45 for the median expression of ISGa, GBPs, and HLAd upon infection, respectively. For the vaccinated group, there was an increase of 3.05, 0.64, and 0.41 for the median expression of ISGa, GBPs, and HLAd upon infection, respectively. The comparison between these two groups reached statistical significance for ISGa (*p* = 0.0077). Therefore, the activation of IFN signaling upon infection was attenuated by prior vaccination, especially for ISGa. Interestingly, a moderate activation of HLAd was observed in this cohort, which was in contrast with the suppression of HLAd in datasets on hospitalized COVID-19 patients. Since this was a cohort of young volunteers enrolled in a clinical trial for vaccination, and the vast majority of the subjects only developed mild symptoms, the immune response was unlikely the same as that in the cohorts of hospitalized patients. Rather, this feature was similar to the observation from the viral challenge experiment (Figure 6A) and the non-hospitalized symptomatic patients (Figure 5A).

One week after the symptom onset, the activation of IFN signaling significantly decreased in both groups. For the unvaccinated group, there was a decrease of 3.49, 1.08, and 0.37 for the median expression of ISGa, GBPs, and HLAd. For the vaccinated group, there was a decrease of 2.54, 0.77, and 0.17 for the median expression of ISGa, GBPs, and HLAd. For ISGa and GBPs, significant deviation from the uninfected state was still observed one week after onset symptoms in the unvaccinated group (seven subjects with ISGa > 3 and five subjects with GBPs > 1).

## 4. Discussion

The COVID-19 pandemic led to millions of death due to the much higher rate of severe diseases compared to other common respiratory infections. Thus, understanding the pathogenesis of severe COVID-19 is crucial for the development of better intervention strategies for COVID-19 and future threats. In our previous work [27], we conducted meta-analysis of blood transcriptome data on COVID-19 and proposed that hypoxia and hypoxia-induced activation of neutrophil degranulation may contribute to severe COVID-19. Due to the stringent cutoff, IFN signaling did not reach statistical significance in the functional enrichment analysis. To explore the role of IFN signaling in the pathogenesis of COVID-19, we conducted targeted analysis of IFN signaling in this work. Based on previous data analysis, the most prominent feature for IFN signaling in viral infection was the activation of type I IFN-stimulated genes. Since the IFN-γ pathway has been widely reported in the literature of COVID-19, we included two sets of marker genes for IFN-γ signaling in addition to one set of marker genes for type I IFN signaling in this targeted analysis of IFN signaling.

High-resolution temporal profiles of IFN signaling upon infection can be revealed in viral challenge experiments. Based on the available information [35], a logical timeline can be revealed. First, viruses will infect local tissues in the respiratory tract and replicate at a fast pace (~2 days). This will induce immune response in local tissues simultaneously, which will be followed by systemic immune response in the circulation (~day 3). Host immune response may lead to some of the symptoms (~day 4). In the next two weeks, innate immunity will gradually come down, and adaptive immunity will gradually build up. For most people with mild COVID-19, this generally signals the end of viral replication and symptoms.

During this whole process, different profiles were observed for the three marker gene sets for IFN signaling despite moderately strong correlation between ISGa and GBPs. ISGa displayed the peak activation on day 4. The activation level stayed high up to day 10. Significant activation was still observable even on day 14. In comparison, more moderate activation was observed for GBPs, with peak expression on day 3 and substantially reduced expression on day 7. Thus, the return to the baseline level was at a much faster pace for GBPs than for ISGa. Overall, the timing for ISGa activation displayed better correlation with viral replication and symptoms. As for HLAd, only minor fluctuation was observed on days 4 and 5, which was consistent with the timing of the peak activation of ISGa.

In hospitalized patients with COVID-19, the profiles of all three marker gene sets changed. First, suppression of HLAd was observed in a significant portion of patients, and the level of suppression was correlated with disease severity. Suppression of GBPs was observed in some of the patients with COVID-19 sepsis and those requiring ECMO support. In addition, the initial activation of ISGa and GBPs may be correlated with viral load in patients with mild COVID-19. As for the temporal dynamics in severe and critical COVID-19, the return to the level of healthy controls was the fastest for GBPs and slowest for HLAd. For some patients, HLAd could not return to the normal level even 28 days after hospital admission. Similar to the activation of neutrophil degranulation, the suppression of HLAd may also be partially induced by hypoxia [42,43]. Although the initial activation of ISGa and GBPs could be involved in restricting viral replication due to the association with viral load, persistent activation of ISGa and GBPs over two weeks may lead to hyper-inflammation and subsequent damage.

It should be noted that HLA-DR+ monocytes has been extensively studied in the context of COVID-19 [44]. The expression of mHLA-DR was significantly lower in COVID-19 patients than that in healthy controls, and the expression levels in severe and critical patients were below the normal range in healthy controls [45]. Severe COVID-19 was characterized by the accumulation of HLA-DR^low^ monocytes [46]. Low mHLA-DR expression was associated with 28-day mortality in critically ill patients with COVID-19 [47]. In young patients with severe COVID-19, low mHLA-DR expression was associated with both clinical severity and probability of survival [48]. Nevertheless, the regulation of relevant proteins and genes may not be limited to monocytes. In fact, our preliminary analysis showed that the features of HLAd as well as GBPs and ISGa were consistently observed in the whole blood (Figure 1, Figure 2, Figure 3, Figure 4, Figure 5 and Figure 6), monocytes (GSE176290) [49], and neutrophils (EGA1004503) [50]. Since HLA-DR is involved in antigen presentation, concerted suppression and slow normalization of HLAd in several blood cell types may contribute to severe COVID-19. The reduction in HLA-DR expression in severe COVID-19 warrants further experimental validation by experts from this field.

Vaccination provides another angle to understand host transcriptional response. Major differences from COVID-19 infection were observed. First, moderate activation of HLAd was consistently observed. Interestingly, moderate activation of HLAd was also observed in some datasets on mild COVID-19 infection (non-hospitalized patients and viral challenge). Second, the activation of ISGa and GBPs was at similar level, which was in contrast with the much higher activation of ISGa in COVID-19 infection. Third, the kinetics were much faster, and the activation was mostly resolved within 4–6 days, whereas it may not be resolved for over two weeks in some patients with COVID-19.

Novel insight may be gained from cross-comparison among natural infection, viral challenge, and vaccination. First, transient activation of all three components at the proper level may be beneficial to the host. Besides restricting viral replication by various mechanisms [51], it may also have a role in inducing adaptive immunity [36]. Second, persistence of the innate immunity over two weeks may be detrimental to the host since it was only observed in hospitalized patients, especially those with severe or critical COVID-19. It has been reported that prolonged activation of IFN signaling in monocytes could lead to the activation of inflammatory cytokines in critical COVID-19 [52]. Activation of type I IFN signaling was even observed in post-COVID-19 condition in a six-month follow-up study of young adults [53]. Another piece of supporting evidence is the substantially reduced activation of ISGa and GBPs in mild breakthrough infection, which is generally with reduced severity. It is possible that the adaptive immunity from the vaccination resulted in the reduced activation of ISGa and GPBs because of the concerted effort from both innate and adaptive immunity. So, it seems to be a virtuous cycle.

The findings from this work may be applied to the disease management of COVID-19. For example, early detection of significant suppression of HLAd is a warning sign of severe disease, and strategies to stimulate HLAd expression within the first week of infection could be beneficial. In addition, persistent activation of GBPs and ISGa over two weeks may be detrimental, and strategies to reduce the expression of GBPs and ISGa at appropriate time could be considered. It has been shown that sustained activation of IFN signaling may delay the development of adaptive immunity in COVID-19 patients [54]. As for the suppression of GBPs or ISGa under certain conditions, it may point to the possibility of genetic defect involving interferon signaling pathways or existence of autoantibodies against interferons [55,56,57,58].

According to the observations presented in the introduction, it seems that HLAd expression could be regulated by IFN-γ. Thus, the main regulator of GBPs expression may not be IFN-γ in COVID-19. Nevertheless, GBPs may still play important roles in COVID-19 that are similar to the roles in host defense against other viruses [51]. GBPs are part of the dynamin family of GTPases. These genes in humans are within a single chromosome cluster. GBPs are known to be involved in anti-bacterial function through lysis of the microbial vacuole. The known anti-viral functions are mainly focused on targeting the life cycle of viruses, including inhibition of viral RNA/protein synthesis and envelope protein processing. GBPs may also target viral proteins for degradation. The regulation of GBPs by IFN stimulation upon viral infection is so far inconclusive.

The findings may also be applied to the field of vaccination. The expression of these gene sets at baseline and one day post vaccination can assist the prediction of vaccination response. Thus, it can identify people who may need to explore other types of vaccines or protections. It can also facilitate the evaluation of overall efficacy of vaccines. Moreover, the data suggest that the temporal kinetics of immune response may not be the same for vaccines delivered through muscle injection and nasal inhalation. This may add another layer of complexity to the development of nasal vaccines, which are intended to reduce infection and spread of the virus [59].

This work has its limitations. First, time is a crucial factor for the understanding of the temporal development of immune response. However, precise information is not available for specific samples in most of the available datasets. Second, the sample size was not ideal for some of the datasets. For example, the dataset for the study of differential immune response upon vaccination had only 10 samples. Thus, caution needs to be taken for the robustness of this specific result. The sampling strategy also varied from one dataset to another. There were many confounding factors that could not be perfectly dealt with in the recruitment of patients and controls (Supplementary Table S1). The effect of age and sex on interferon signaling was analyzed in this work. However, factors such as pre-existing diseases may also have some impact on IFN signaling, which was generally unavailable for individual subjects. There are also other hidden confounding factors, such as unreported batch information. An ideal scenario is large cohorts with baseline information and high-frequency sampling upon symptom onset. It would also be helpful to have simultaneous measurement of some relevant factors in the serum. Additionally, gene expression is intrinsically dynamic. These may be the sources of some variations between studies. Despite all of these caveats, highly consistent observations from multiple studies clearly demonstrated the robustness of the activation/suppression pattern of the marker genes for IFN signaling.

To examine the temporal profiles of IFN signaling, three sets of marker genes were selected for in-depth investigation in this work. Unlike a simple activation or suppression scenario, complex features were revealed in the data analysis (Supplementary Figure S4). Marker genes from different pathways demonstrated distinctive features. Distinctive features were also observed in different marker gene sets from the same pathway. The variations included the direction and scale of regulation, the pace of return to the level of reference, and the correlation with disease severity and viral load. Apparently, these marker gene sets are all regulated through different mechanisms. Although a complex scenario emerged from this analysis, the IFN signaling is far more complex than what has been presented here with the three marker gene sets. For example, two other marker genes for IFN-γ signaling (*TAP1* and *TAP2*) close to the HLA-D gene cluster displayed very strong correlation with the expression of the GBP gene cluster in our preliminary analysis. Thus, the intricate regulation of IFN signaling deserves more comprehensive investigation in the future.

## 5. Conclusions

In this work, regulation of IFN signaling in COVID-19 was examined using three marker gene sets. The inclusion of natural infection, viral challenge, and vaccination made it possible to understand the roles of IFN signaling in a more comprehensive way. Distinctive temporal profiles of IFN signaling were observed in natural infection, viral challenge, and vaccination. The observations from this work may be helpful for monitoring the disease course and vaccination response. It may also provide novel insights into the development of innovative intervention strategies and better vaccines.

## Figures and Tables

**Table 1:** Transcriptome datasets for the examination of IFN signaling upon COVID-19 infection or vaccination.

Accession ID	Condition	Sample Numbers	Ref.
GSE152641	COVID-19 infection Hospitalized patients	62 patients + 24 controls, 23 patients intubated	[23]
GSE243217	COVID-19 infection Sepsis patients	57 sepsis + 15 controls 35 COVID-19, 22 bacterial	[24]
Mendeley 8wxhhykfnh.2	COVID-19 infection Severity and time	103 COVID-19 + 27 controls + 22 flu patients	[25]
Zenodo 6120249	COVID-19 infection Severity and time	91 COVID-19 + 10 controls + 42 sepsis patients	[26]
GSE213313	COVID-19 infection Longitudinal and severity	83 COVID-19, 11 controls, T1, T2, and T3	[27]
EGAS00001005332	COVID-19 infection Longitudinal and severity	158 COVID-19 + 22 controls Day 0 and day 28	[28]
E-MTAB-12993	SARS-CoV-2 challenge Longitudinal	374 blood samples, Day 0 to day 28	[29]
GSE190001	mRNA vaccination Longitudinal	23 subjects, 11 time points 226 samples	[30]
GSE247401	mRNA or mixed vaccination Second/third dose	17 subjects, 4 time points 50 samples	[31]
GSE199750	mRNA or mixed vaccination First/second/third dose	86 subjects, 4 time points 260 samples	[32]
GSE246525	mRNA vaccination High/low response	10 subjects, 4 time points 38 samples	[31]
GSE250023	mRNA vaccination SLE patients, Responder/non-responder	18 subjects, 5 time points 75 samples	[33]
GSE228839	Breakthrough infection w/o prior vaccination	71 subjects, 3 time points 122 samples	[34]

## Data Availability

All of the datasets analyzed in the current work are publicly available. The transcriptome datasets are available at GEO (gene expression omnibus, https://www.ncbi.nlm.nih.gov/geo/, accessed on 5 June 2025) and other sources as specified. For more details, please refer to the Methods Section and Table 1.

## Supplementary Materials

The following supporting information can be downloaded at https://www.mdpi.com/article/10.3390/v17081060/s1. Figure S1: Comparison of ISGs in COVID-19 patients with different age or sex; Figure S2: Regulation of ISGs in COVID-19 patients at different time and severity levels; Figure S3: Regulation of ISGs in COVID-19 patients at different time and severity levels; Figure S4: Temporal profiles of interferon signaling in natural infection, viral challenge, and vaccination; Table S1: Demographic information for transcriptome datasets on COVID-19 infection or vaccination.
