# Supplementary material for: Distinctive Temporal Profiles of Interferon-Stimulated Genes in Natural Infection, Viral Challenge, and Vaccination
Source: Viruses. 2025 Jul 29;17(8):1060. doi: 10.3390/v17081060 (PMC12390552; doi:10.3390/v17081060)
Supplement: Supplementary file 1 [file viruses-17-01060-s001.zip › Supp Figures S1-S4.pdf]

A

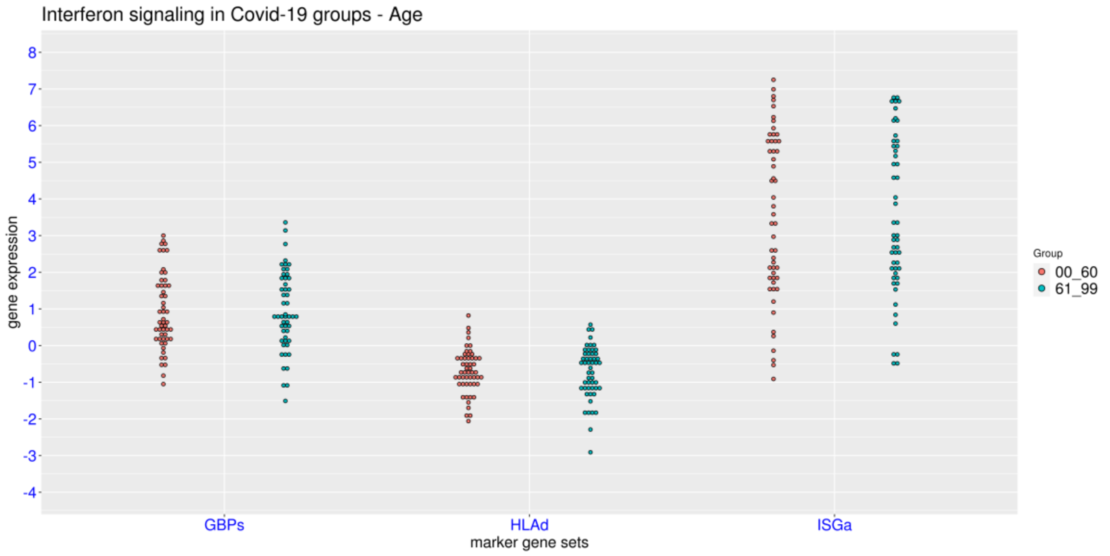

B

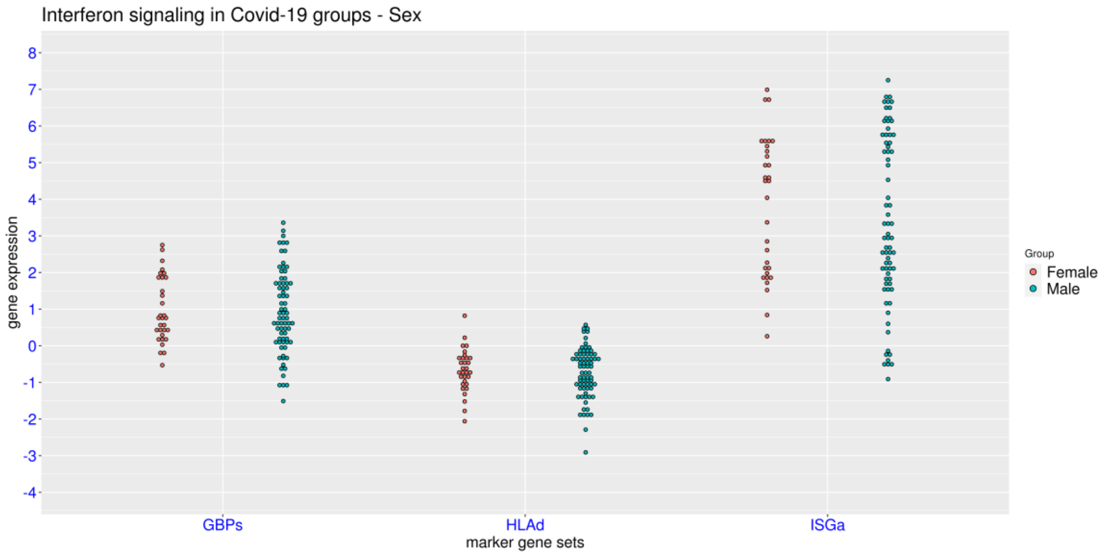

Supplementary Figure S1. Comparison of interferon-stimulated genes in Covid-19 patients with different age or sex (dataset Mendeley 8wxhhykfnh.2). **A**, upper panel. Comparison of interferon signaling in two age groups (0-60 and 61-99) **B**, lower panel. Comparison of interferon signaling in males and females.

A

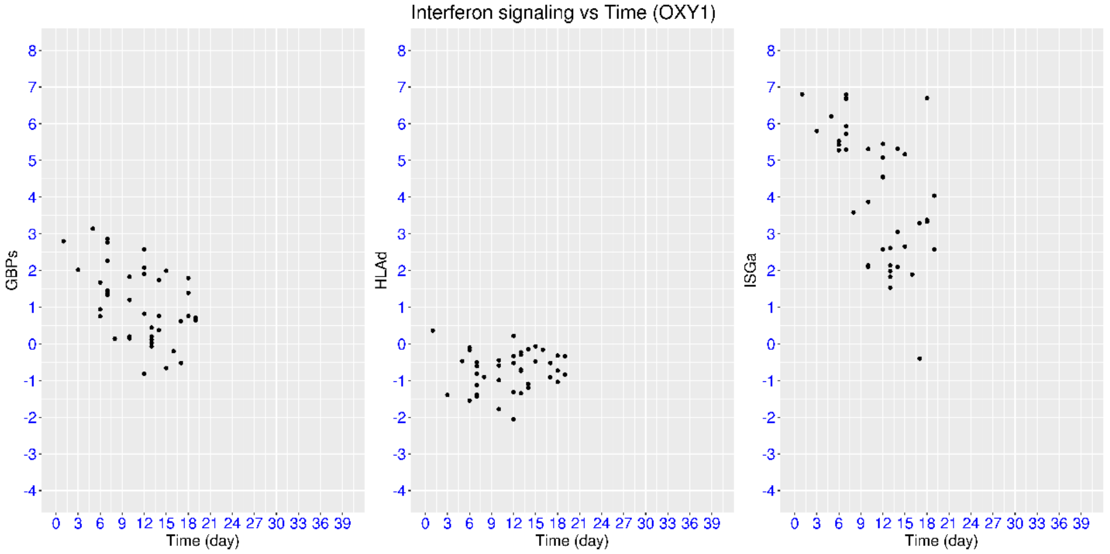

B

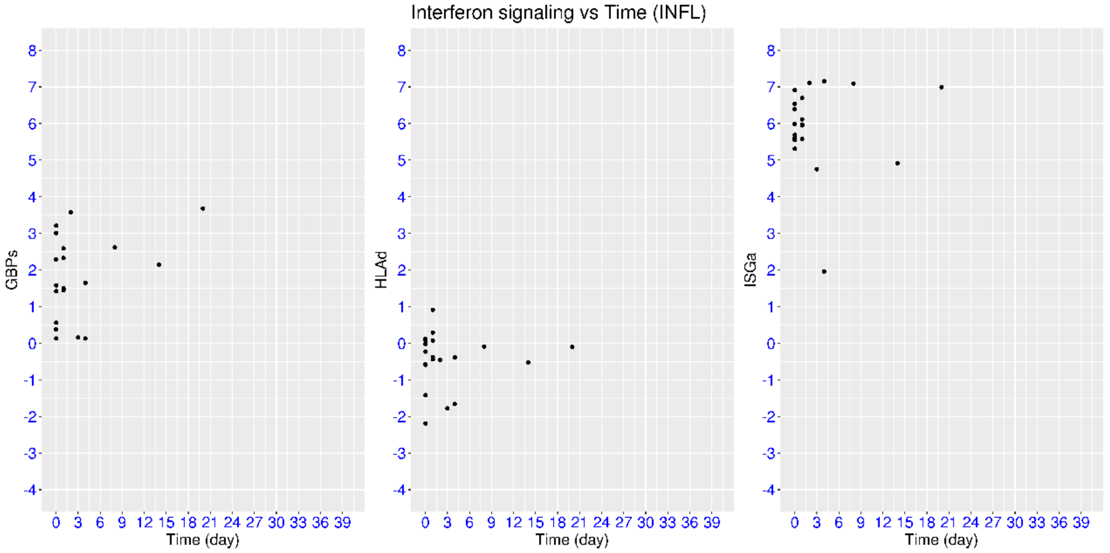

Supplementary Figure S2. Regulation of interferon-stimulated genes in Covid-19 patients at different time and severity levels (dataset Mendeley 8wxhhykfnh.2). **A**, upper panel. Regulation of interferon signaling at different time (days from onset symptoms) in the OXY1 group (supplementary oxygen required) **B**, lower panel. Regulation of interferon signaling at different time (days from onset symptoms) in the INFL group (patients with mild flu).

A

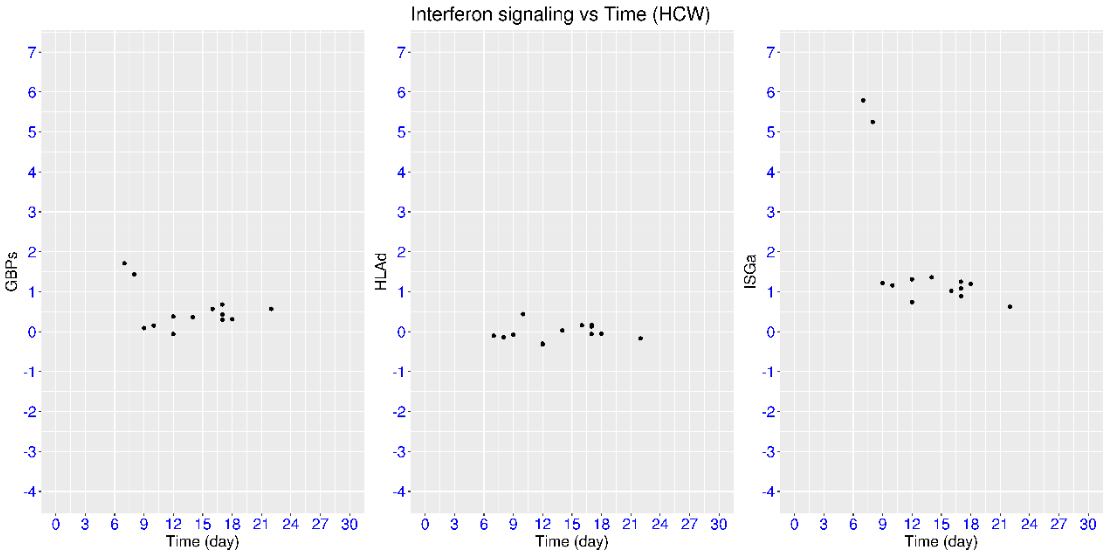

B

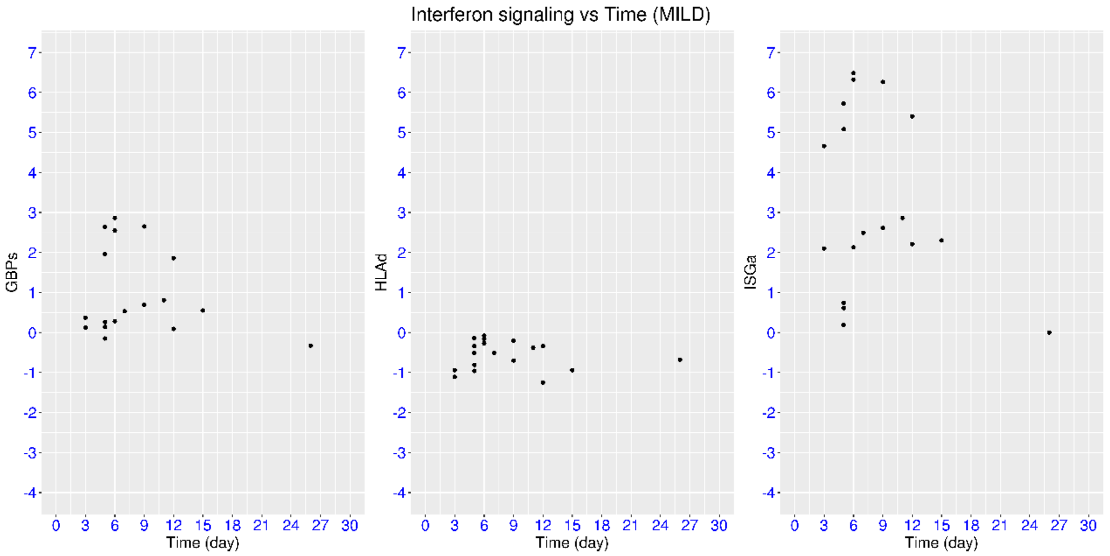

Supplementary Figure S3. Regulation of interferon-stimulated genes in patients with Covid-19 at different time and severity levels (dataset Zenodo 6120249). **A**, upper panel. Regulation of interferon signaling at different time (days from onset symptoms) in the HCW group (health care workers) **B**, lower panel. Regulation of interferon signaling at different time (days from onset symptoms) in the mild group (no oxygen requirement).

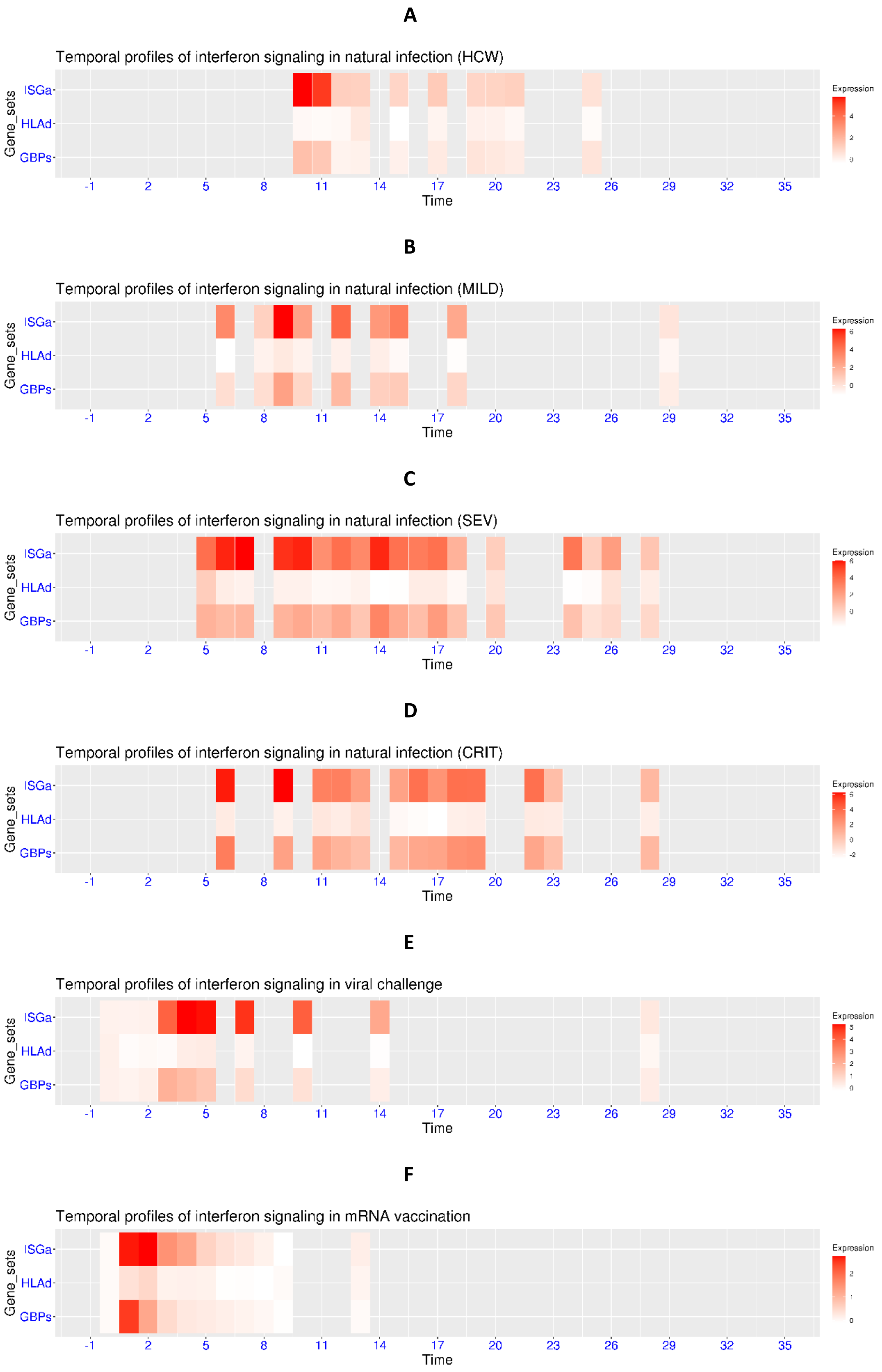

Supplementary Figure S4. Temporal profiles of interferon signaling in natural infection, viral challenge and vaccination. **A-D**, natural infection (dataset Zenodo 6120249) with four patient groups, including recovering health care workers (HCW),

mild (MILD), severe (SEV) and critical (CRIT) patients with Covid-19. The median expression values at each time point were used in the plotting. The plots may look noisy because of the small sample size for most of the time points. To align better with the viral challenge dataset, the time was right-shifted three days (please refer to the main text for the reason). **E**, viral challenge (dataset E-MTAB-12993). The median expression values at each time point were used in the plotting. **F**, mRNA vaccination (dataset 190001). The median expression values at each time point were used in the plotting. To align better with the viral challenge dataset, the time was left-shifted one day due to the different definition of day 0.
