# Supplementary material for: Distinctive Temporal Profiles of Interferon-Stimulated Genes in Natural Infection, Viral Challenge, and Vaccination
Source: Viruses. 2025 Jul 29;17(8):1060. doi: 10.3390/v17081060 (PMC12390552; doi:10.3390/v17081060)
Supplement: Supplementary file 1 [file viruses-17-01060-s001.zip › Supp Table S1.pdf]

Supplementary Table S1. Demographic information for transcriptome datasets on Covid-19 infection or vaccination.

Accession ID	Age	Gender	Time (days from onset)
GSE152641	Median 61<br>IQR: 52-70	65% male	Median 6<br>IQR: 4-8
GSE243217	Median 72<br>IQR: 59-76	68.6% male	N/A
Mendeley<br>8wxhhykfnh.2	N/A	70.0% male	Median 12<br>Range: 1-37
Zenodo<br>6120249	Mild: 73 (21-95)<br>SEV: 71 (22-95)<br>CRIT: 57 (31-78)	Mild: 42% male<br>SEV: 62% male<br>CRIT: 72% male	Mild: 6 (3-26)<br>SEV: 9 (1-37)<br>CRIT: 13 (3-36)
GSE213313	Critical: 62.1 (8.5)<br>Severe: 54.7 (15.4)	Critical: 65% male<br>Severe: 60% male	Critical: 9.6 (2.2)<br>Severe: 9.2 (2.8)
EGAS00001005332	A: 32.9 (12.7)<br>B: 36.0 (11.8)<br>C: 58.0 (16.9)<br>D: 64.4 (15.1)<br>E: 57.0 (14.9)	A: 22.2% male<br>B: 22.5% male<br>C: 54.3% male<br>D: 64.9% male<br>E: 75.0% male	A: N/A<br>B: 6.5 (2.9)<br>C: 11.4 (6.7)<br>D: 10.6 (8.1)<br>E: 24.6 (14.3)
E-MTAB-12993	18-30<br>Healthy volunteers	N/A	N/A
GSE190001	38 (29-57)	34.8% male	N/A
GSE247401	N/A	20% male	N/A
GSE199750	37 (19-68)	30.6% male	N/A
GSE246525	N/A	All female	N/A
GSE250023	38 (22-49)	All female	N/A
GSE228839	Vac: 38 (19-65)<br>Mock: 36 (20-55)	Vac: 35.7% male<br>Mock: 23.1% male	N/A

Note: the demographic information was for the hospitalized Covid-19 patient group in each dataset on natural infection. The format A (B-C) refers to median (range), while the format A (B) refers to median (SD). The dataset GSE213313 were generated on a microarray platform, all other datasets were generated on RNA-Seq platforms.
